# Familial Abdominal and Intestinal Lipomatosis Presenting with Upper GI Bleeding

**DOI:** 10.1155/2015/123723

**Published:** 2015-06-04

**Authors:** Yilmaz Bilgic, Hasan Baki Altinsoy, Nezahat Yildirim, Ozkan Alatas, Burhan Hakan Kanat, Abdurrahman Sahin

**Affiliations:** ^1^Department of Gastroenterology, Faculty of Medicine, Inonu University, Malatya, Turkey; ^2^Department of Radiology, Elazig Training and Research Hospital, Elazig, Turkey; ^3^Department of Pathology, Elazig Training and Research Hospital, Elazig, Turkey; ^4^Department of General Surgery, Elazig Training and Research Hospital, Elazig, Turkey; ^5^Department of Gastroenterology, Elazig Training and Research Hospital, Elazig, Turkey

## Abstract

Although lipomas are encapsulated benign tumors, systemic lipomatosis defines infiltrative nonencapsulated tumors resembling normal adipose tissue. Abdominal lipomatosis and intestinal lipomatosis are different clinicopathological entities with similar clinical symptoms. We describe here a case presenting with upper gastrointestinal bleeding from eroded submucosal lipoma at duodenum secondary to intestinal lipomatosis and abdominal lipomatosis.

## 1. Introduction

Multiple systemic lipomatosis (MSL) is a rare disorder with unknown etiology, characterized by the accumulation of nonencapsulated adipose tissue at face, head, neck, upper and lower extremities, trunk, abdominal cavity, and pelvis [1]. Involvement of gastrointestinal (GI) tract and abdominal cavity is very rare. Abdominal and intestinal involvements present with abdominal pain, obstruction, intussusception, and GI bleeding. Abdominal lipomatosis might also cause distension, via intermittent obstruction and altering bowel transit time. Intestinal lipomatosis refers to having multiple submucosal lipomas at small intestine and colon. We describe here a case with bleeding duodenal lipoma related to intestinal lipomatosis and diffuse abdominal lipomatosis that is deforming stomach and proximal small bowel loops.

## 2. Case Presentation

A 35-year-old man was admitted to emergency department with one-day history of hematemesis and melena after taking nonsteroidal anti-inflammatory drug. He had no medical illness. He denied smoking, alcohol consumption. The height and weight of patient were 178 cm and 68 kg, respectively (body mass index: 21 kg/cm^2^). On physical examination, blood pressure was 120/70 mmHg and pulse rate 86/min. There was a 2 cm mobile painless submucosal nodular lesion on the left neck. His abdomen was soft with no tenderness, rebound, mass lesion, or ascites. The patient was promptly given intravenous crystalloid and colloid fluid replacement; parenteral proton-pump inhibitor was administered. Initial laboratory values were as follows: leukocyte: 5800/mm^3^, hemoglobin: 14.8 g/dL, platelet: 347.000/mm^3^, prothrombin time: 12 sec, and INR: 1.05. Biochemical parameters were normal.

**Figure 1:**
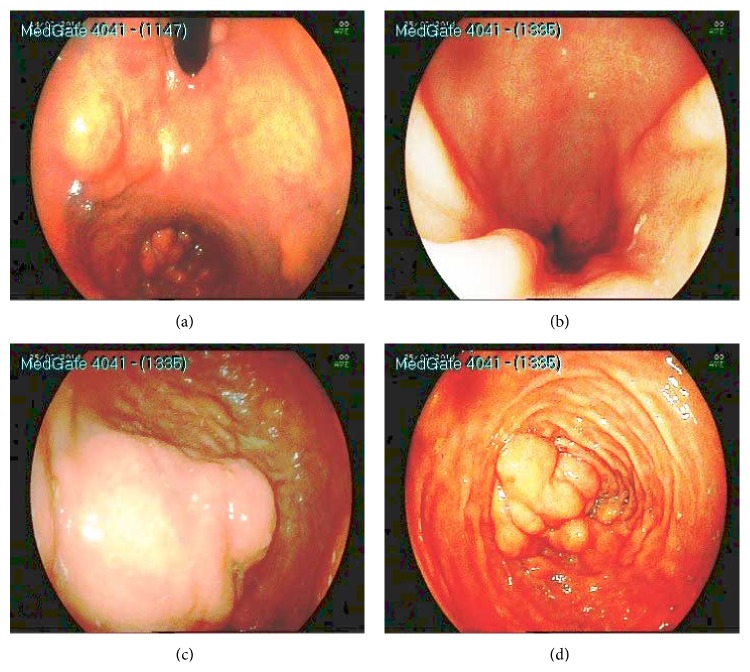
Endoscopic view of submucosal lesions: (a) fundus at retroflection position, (b) antrum and pylorus, (c) bulb and bleeding submucosal lesion, and (d) second part of duodenum.

**Figure 2:**
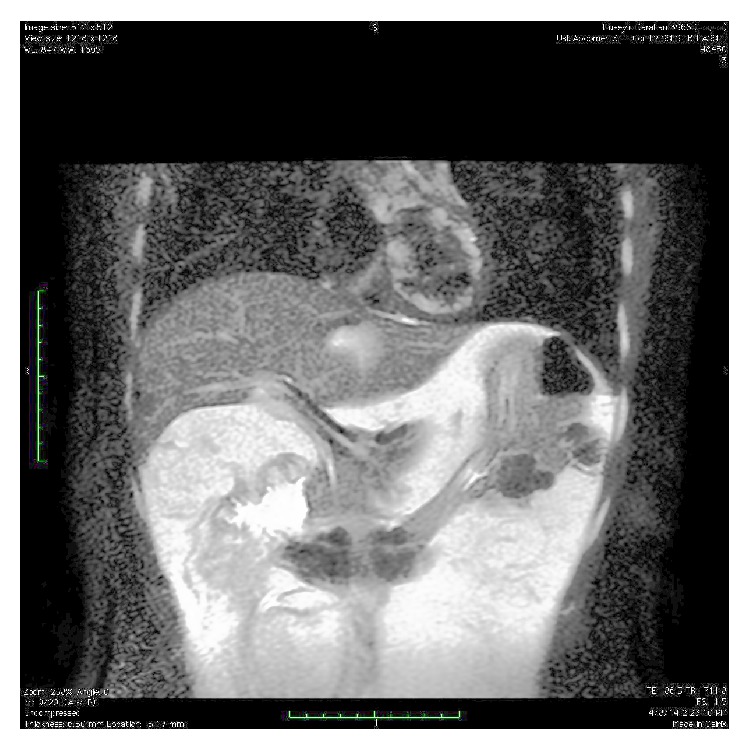
Coronal T2 weighted MR imaging of diffuse abdominal lipomatosis, luminal narrowing at distal part of stomach and bulb secondary to diffuse lipomatosis and antral submucosal lipoma with deplased intestinal loops.

**Figure 3:**
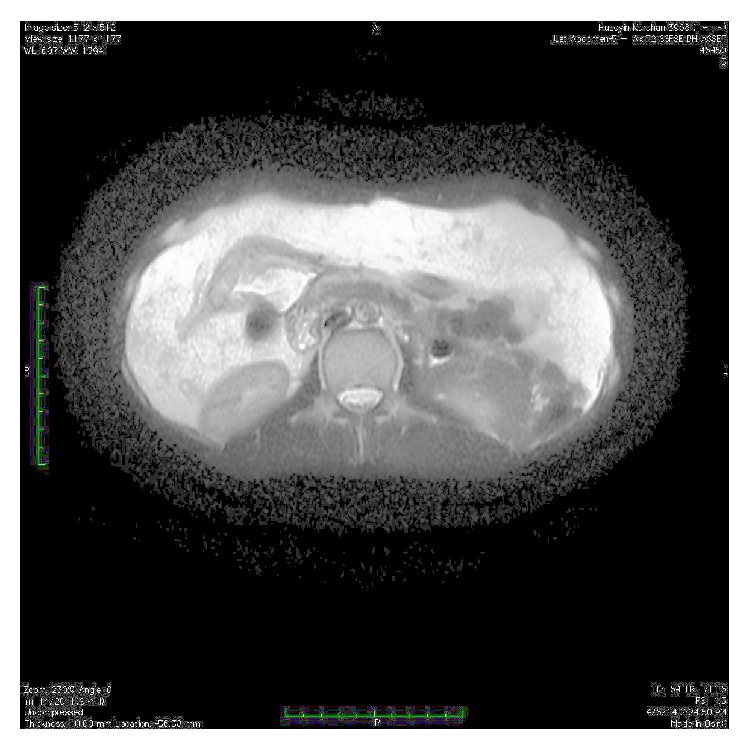
Axial T2 weighted MR imaging of abdominal cavity with diffuse abdominal lipomatosis, luminal obliteration of antrum and distal part of corpus due to lipoma and clustered intestinal loops at the right upper part of intestinal cavity.

**Figure 4:**
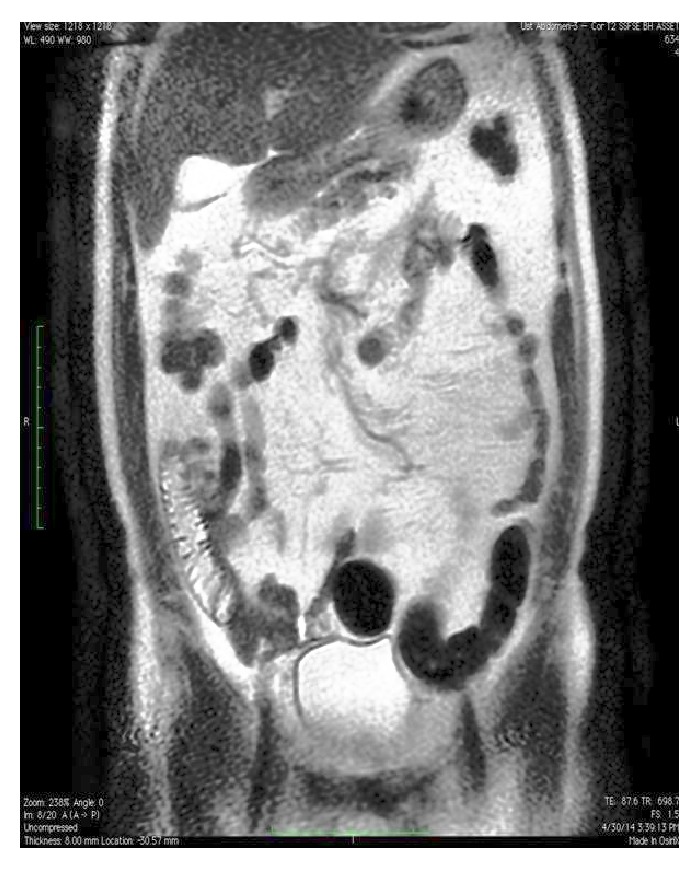
Coronal T2 weighted MR imaging with diffuse abdominal lipomatosis of the father of index case.

Upper gastrointestinal endoscopy revealed gastric and duodenal multiple submucosal mobile lesions with narrowing of antrum and constant deformation of duodenum. It was unable to proceed to the second part of duodenum with endoscope (Figure 1). Colonoscopic examination was normal. Abdominal magnetic resonance imaging (MRI) showed that diffuse lipomatosis of abdominal cavity extending to the mediastinum (Figure 2) and obliteration at the distal part of stomach, bulb, and proximal duodenum due to submucosal lipomatous lesions with deplased small intestine loops to the right upper abdomen (Figure 3). Laboratory investigation showed fasting glucose 68 mg/dL, fasting insulin 1.18 mg/dL, triglyceride 53 mg/dL, low density lipoprotein 62 mg/dL, and high density lipoprotein 47 mg/dL. His father similarly has mobile nontender lesions at neck and right upper extremity. However, submucosal lesions were not detected on UGIE examination. Abdominal MRI of his father showed similarly lipomatous tissue increase in abdominal cavity (Figure 4). Endoscopic biopsy of submucosal polypoid lesion was diagnosed as lipoma and trucut biopsy of diffuse mass lesion filling abdominal cavity was consistent with fibroadipose tissue.

## 3. Discussion

Multiple symmetrical lipomatosis affects mostly white men between 25 and 60 years of age. It may occur sporadically or familiarly [2]. Diffuse abdominal lipomatosis is a variant of MSL characterized by massive enlargement of abdomen by collection of large nonencapsulated lipomas [3]. This rare disease might be associated with dyslipidemia (high triglyceride, high HDL), insulin resistance, hyperuricemia, macrocytic anemia, and peripheral neuropathy [2]. Several etiological factors are assumed in the development of lipomatosis such as embryonic displacement of adipose tissue, congenital predisposition, degenerative disease with disturbance of fat metabolism, postchemotherapeutic fat deposition, chronic inflammation such as chronic inflammatory bowel disease, low-grade infection, and hamartomatous syndromes and alcohol [4]. Alcohol consumption is common coexisting factor that causes folate deficiency, macrocytic anemia and promote lipomas through effects on adiposities [1].

In MSL, adipocytes have increased lipoprotein lipase activity and a defect in adrenergic lipolysis. Adipogenesis in MSL is not a consequence of energy excess but it is an active hyperplastic proliferation of subcutaneous adipose tissue. This kind of behavior of some adipocytes in several subcutaneous areas in MSL suggests that the energy unrelated adipogenesis could contribute to the expansion of adipose tissue [5]. Chen et al. demonstrated abdominal and subcutaneous adipose tissue accumulation did not induce glucose and lipid metabolism dysfunction in MSL [6]. Our patient supports this finding, as not to have dyslipidemia, glucose intolerance, or alcohol consumption. Furthermore, father of the patient had lipoma on the neck and increased fat tissue in the abdominal cavity at MRI, similarly. Familial tendency to the accumulation of fat in the abdominal cavity might be the cause of MSL in this case.

Diffuse abdominal lipomatosis and intestinal lipomatosis are two distinct clinical entities. Abdominal lipomatosis refers to massive infiltrative nonencapsulated fat accumulation. However, intestinal lipomatosis defines the presence of multiple lipomas. Gastrointestinal lipomas occur predominantly in the large intestine (especially right sided) and then decreasing prevalence in the small bowel, stomach, and esophagus [7]. We found multiple gastric and duodenal lipomas at upper GI endoscopic examination. We did not detect lipomas via colonoscopic examination or radiologically at distal part of small intestine and colon. On the contrary of the literature, our patient has lipomas at stomach and small intestine. Abdominal lipomatosis and intestinal lipomatosis present together in our patient. Abdominal lipomatosis might be asymptomatic as in the father of index case.

Intestinal lipomatosis related GI bleeding revealed coexistence of these two very rare diseases. In patients presenting with obstructive symptoms, paroxysmal abdominal pain, or GI hemorrhage, endoscopic treatment including endoscopic submucosal dissection, endoscopic snare resection, or surgical intervention might be definitive treatment [8, 9]. With conservative management, bleeding stopped spontaneously and did not recur. Although imaging studies demonstrated compression of upper small bowel and stomach, the patient did not suffer from distension, intermittent abdominal pain, or other obstructive symptoms. This patient should be followed closely for obstructive symptoms. In case of obstructive findings, surgical resection is the treatment of choice.
